# The significance of PAK4 in signaling and clinicopathology: A review

**DOI:** 10.1515/biol-2022-0064

**Published:** 2022-06-20

**Authors:** Xinbo Yu, Changwei Huang, Jiyuan Liu, Xinyu Shi, Xiaodong Li

**Affiliations:** The First Clinical College, China Medical University, Shenyang, Liaoning Province 110122, China; Department of Cell Biology, Key Laboratory of Cell Biology, National Health Commission of the PRC and Key Laboratory of Medical Cell Biology, Ministry of Education of the PRC, China Medical University, Shenyang, Liaoning Province 110122, China; The Second Clinical College, China Medical University, Shenyang, Liaoning Province 110122, China

**Keywords:** PAK4, signaling, clinicopathology, cancer

## Abstract

P21-activated protein kinases (PAKs) are thought to be at the center of tumor signaling pathways. As a representative member of the group II PAK family, P21-activated protein kinase 4 (PAK4) plays an important role in the development of tumors, with several biological functions such as participating in oncogenic transformation, promoting cell division, resisting aging and apoptosis, regulating cytoskeleton and adhesion, as well as suppressing antitumor immune responses. PAK4 is also crucial in biological processes, including the occurrence, proliferation, survival, migration, invasion, drug resistance, and immune escape of tumor cells. It is closely related to poor prognosis and tumor-related pathological indicators, which have significant clinical and pathological significance. Therefore, this article offers a review of the structure, activation, and biological functions of PAK4 and its clinical and pathological importance. This overview should be of assistance for future research on PAK4 and tumors and provide new ideas for tumor treatment and prognostic evaluation of patients.

## 1 Introduction

The occurrence and development of tumors involve complex processes and multiple mechanisms. Ongoing research on tumors has led to increased recognition of the important role of P21-activated protein kinases (PAKs) in the occurrence and development of tumors. P21-activated protein kinase 4 (PAK4) is a representative member of the group II PAK family and mediates almost all biological behaviors of tumors at the cell level, from oncogenic transformation [1] to continuous proliferation [2] and from the tenacity of cancer cells [3] to their metastasis [2], drug resistance [4], and immune escape [5]. As a potential tumor biomarker, PAK4 has great clinical and pathological significance in the evaluation of prognostic and pathological indicators [6]. Therefore, it has become a popular research topic in tumor signal transduction and treatment in recent years. Here, we review the structure, activation, and biological function of PAK4 as well as its clinical and pathological importance to provide context for future preclinical and clinical research on PAK4 and tumors.

## 2 Structure of PAK4

The PAK serine/threonine kinase family consists of six members. These members are divided into two groups according to their structural and activation characteristics: group I (PAK1–3) and group II (PAK4–6). Both groups of PAKs share a similar structure: an amino-terminal p21-binding domain (PBD) which can bind Rho GTPases (Cdc42/Rac), and a highly conserved serine/threonine kinase domain at the carboxy-terminal. The group I PAKs have two proline-rich regions (PXXP) in front of the PBD. Moreover, an autoinhibitory domain (AID) behind the PBD acts with the PBD as a dimer. There is also a PAK-interacting exchange factor (PIX) binding domain that binds PIX between the AID and the serine/threonine kinase domain and a Gβγ binding domain behind the kinase domain. Group II PAKs also have a p21-binding domain and a kinase domain, which are slightly different from group I. The group II PAKs were believed not to contain an AID, but later studies have found that they have a sequence similar to the AID. However, this sequence does not overlap with PBD but is present on its own behind the PBD. Meanwhile, some models suggest that the AID-like domain beside the PBD, which keeps PAK4 inactive, is an autoinhibitory pseudosubstrate domain (PSD) [7,8,9,10].

**Figure 1:**
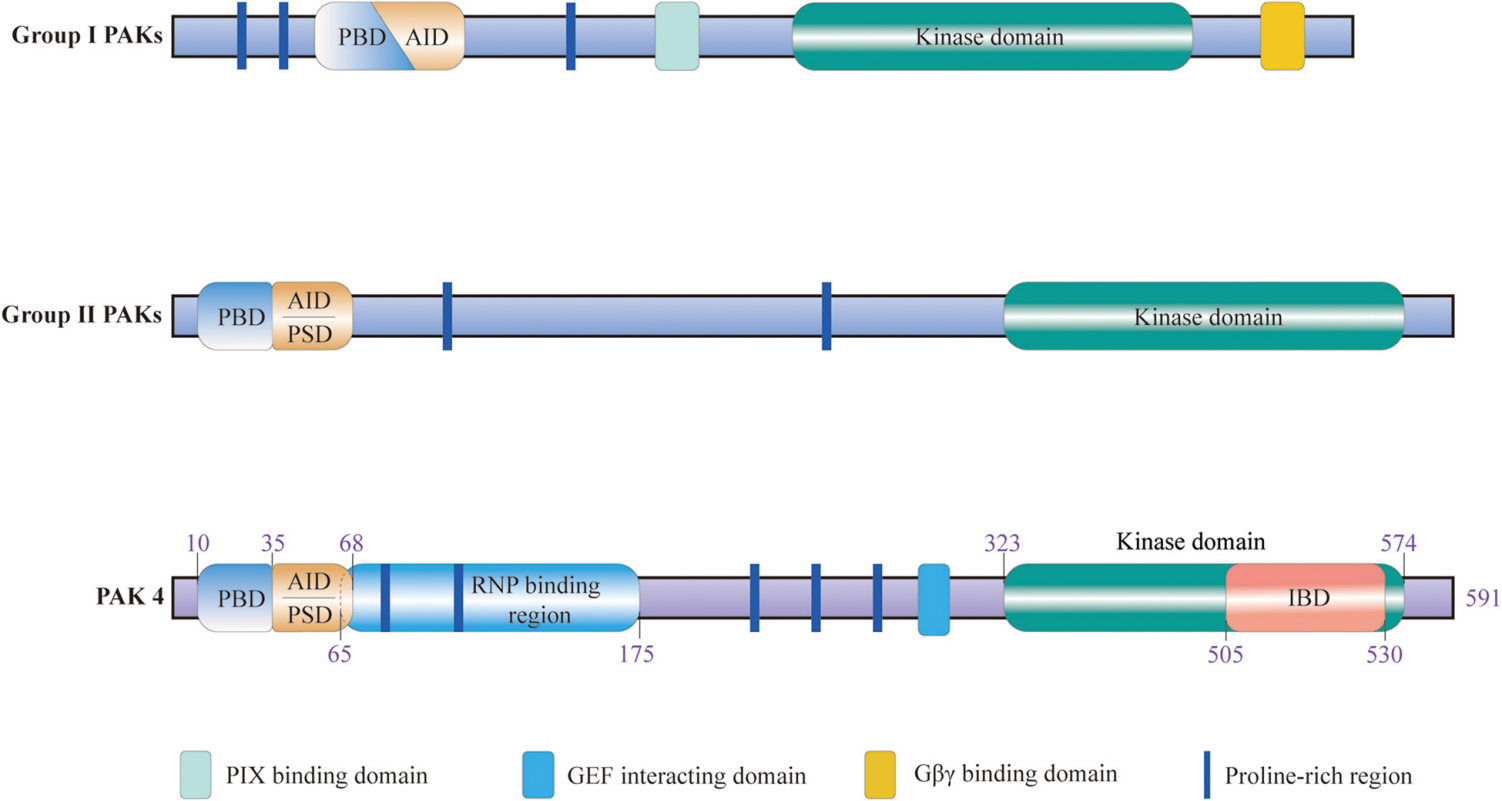
Structure of group I and group II PAKs. All PAKs have PXXP, an amino-terminal PBD, and a carboxy-terminal kinase domain. Group I PAKs have an extra PIX binding domain, Gβγ binding domain, and AID behind the PBD, which acts with the PBD as a dimer. Group II PAKs have an additional AID-like domain (some models suggest it is PSD) that exists alone rather than forms a complex with PBD. PAK4 structure is highlighted. The length of PAK4 is 591aa. Besides its PBD(10–35aa), AID-like domain/PSD(35–68aa), kinase domain (323–574aa), and PXXP, PAK4 also has a RNP binding domain (65–175aa), GEF interacting domain, and IBD (505–530aa). PXXP: proline-rich regions; PBD: p21-binding domain; PIX: PAK interacting exchange factor; AID: autoinhibitory domain; PSD: pseudosubstrate domain; RNP: ribonucleoprotein; GEF: guanosine exchange factor; IBD: integrin-binding domain; aa: amino acid.

PAK4 is the first member found in the group Ⅱ PAKs and consists of 591 amino acids (aa). In PAK4, PBD is located at 10–35aa. As in other group II PAKs, the AID-like sequence of PAK4 does not overlap with the PBD as the AID of group I PAKs does but plays its role independently of the PBD sequence. Moreover, a region behind the PBD interacts with the ribonucleoprotein [11]. The serine/threonine kinase domain is located at 323–574aa in PAK4. An integrin-binding domain (IBD) is located in the 505–530aa of PAK4, in the posterior part of the serine/threonine kinase domain, which can bind to some integrins [12,13]. Although this structure is thought to exist in other kinds of PAKs, it has not yet been reported [9]. The kinase domain of PAK4 forms an αC helix, a glycine-rich loop, and some activated fragments spatially, but most of these structures are disordered and very flexible [14]. In addition to the three main structures (the PBD, the AID-like sequence, and the serine/threonine kinase domains), there are several PXXP between the AID-like sequence and the kinase domain, as well as a guanosine exchange factor (GEF) interacting domains behind the PXXP (Figure 1) [8,9,10].

## 3 Regulation of PAK4 kinase activity

**Figure 2:**
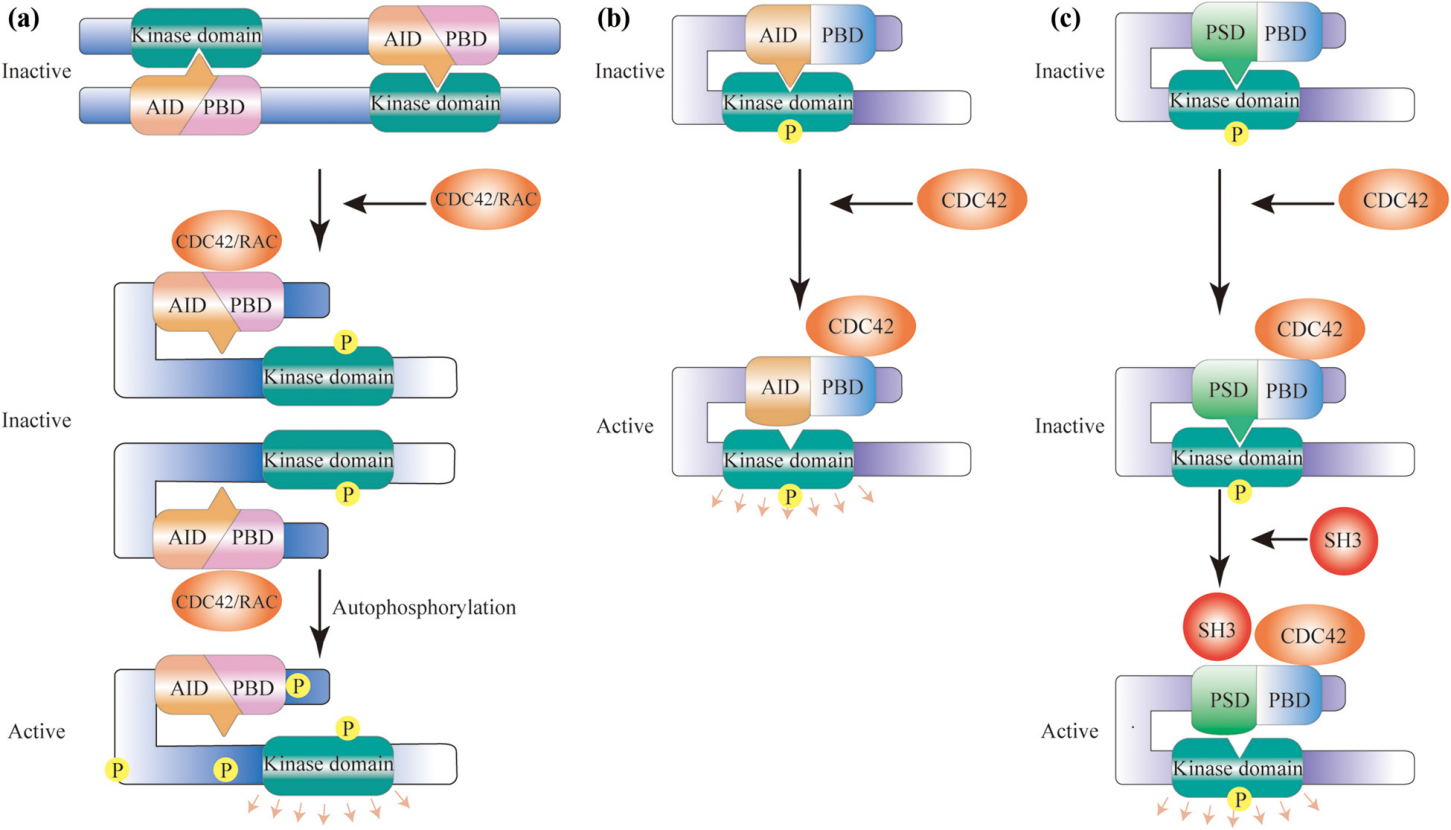
Activation of group Ⅰ and group Ⅱ PAKs. (a) The activation of group I PAKs: Two group I PAKs monomers constitute a homodimer. The AID of one monomer is inserted into the kinase domain of the other and inactivates it. The combination of PBD and CDC42/RAC can relieve the auto-inhibition state, leading to the autophosphorylation and activation of the PAKs. (b) Model 1 for the activation of PAK4: The PAK4 monomer remains inactive due to the binding of the kinase domain and the AID-like sequence, but can be activated by binding with CDC42. (c) Model 2 for the activation of PAK4: The interaction of PSD with the kinase domain can inhibit PAK4 activity. The activation of PAK4 involves two steps. CDC42 binds to PBD first to relocate PAK4 in cells. Subsequently, SH3 proteins bind to PSD, resulting in the activation of PAK4. P: phosphorylation; AID: autoinhibitory domain; PBD: p21-binding domain; PSD: pseudosubstrate domain; SH3: src homology 3.

Compared to research on group II PAKs, that on the group I PAKs has been more thorough. The regulation mechanism of group I PAKs is trans-auto-inhibition [15]. In the inactive state, two group I PAKs monomers fold into a homodimer in a head-to-tail manner [16]. The AID of one monomer is inserted into the kinase domain of the other [17]. This interaction inhibits autophosphorylation of the PAKs and activation of the kinase domain. Activation of group I PAKs mainly depends on the combination of PBD and CDC42/Rac. A series of conformational changes occur after their binding, and the homodimer state is no longer maintained, so the inhibitory effect of the AID on the kinase domain is reduced. The PAKs are autophosphorylated at multiple sites, switching it from an inactive state to an active state [18,19] (Figure 2a). Group I PAKs can also be regulated by phosphatidylinositol, proteins that contain src homology 3 (SH3) domains, 3-phospho-inositide-dependent kinase-1, PIX, and other mechanisms [20,21,22,23].

The activation mechanism of group II PAKs is different from that of group I PAKs. For group I PAKs and most protein kinases, phosphorylation of the activation loop is related to kinase activity [24]. However, Ser474 on the activation loop of PAK4 is constitutively autophosphorylated [25]. The phosphorylation of the activation loop is not related to kinase activity, so the change in activity may depend on conformational changes [26]. Presently, there are two clear activation mechanisms of PAK4. One study showed that activation is dependent on the conformational changes of PAK4 mediated by CDC42. PAK4 exists as a monomer in the inactive state and remains inactive due to the binding of the kinase domain and the AID-like sequence. Also, PAK4 is activated when active Cdc42 binds to it and causes conformational change [24] (Figure 2b). Another study showed that activation of PAK4 is dependent on the reduction of PSD autoinhibition mediated by SH3 proteins. The interaction of PSD with the kinase domain can keep PAK4 in an inactive state [14]. During the activation process of PAK4, CDC42/RAC binds to it first, not activating the kinase, but reorienting it [27]. Subsequently, PSD binds to SH3 proteins, resulting in the reduction of auto-inhibition and kinase activation [14] (Figure 2c). Group II PAKs can also be activated through other mechanisms. For example, hepatocyte growth factor (HGF) can enhance the kinase activity of PAK4 in MDK cells [28].

## 4 PAK4 and tumor occurrence or development

### 4.1 PAK4 and oncogenic transformation

PAK4 is closely related to the occurrence of tumors. It participates in oncogenic transformation and mediates pre-tumor biological effects such as anchorage-independent growth [29], epithelial-mesenchymal transition (EMT) [30], and polarization loss [31], and thus is a key transduction factor of oncogenic signals. PAK4 undergoes gene amplification in a variety of tumor cells. Its gene is located on chromosome 19q13 (which is associated with tumorigenesis and frequently amplified) and is regarded as an amplification target in a variety of tumors such as pancreatic cancer (PC) and oral squamous cell carcinoma (OSCC) [29,32,33]. Amplification of the PAK4 gene leads to overexpression of PAK4, which is often consistent with oncogenic transformation. Anchorage-independent growth is an important feature of cell oncogenic transformation [34]. One study showed that activated PAK4 induces anchorage-independent growth of mouse fibroblasts and plays a key role in Ras-driven cell transformation [29]. Parvin Beta (ParvB) inhibits integrin-linked kinase, which is associated with anchorage-independent growth [35]. Through next-generation sequencing, it was found that overexpression of PAK4 in mouse mammary epithelial cells (iMMECs) showed a downregulation of ParvB gene expression [30], which also suggests a positive role for PAK4 in anchorage-independent growth. EMT gives cells the ability to metastasize and invade and is considered to be a key step in the oncogenic transformation of cells [36]. Forkhead Box C2 (FoxC2) is a transcription factor that regulates cell migration, and its expression increases during EMT [37]. Overexpression of PAK4 in iMMECs was correlated with an increase in FoxC2 gene expression, suggesting that PAK4 promotes EMT [30]. Loss of polarization is closely related to oncogenic transformation, and the loss of apical polarization of epithelial cells is usually followed by cancer [38]. The overexpression of PAK4 leads to disordered cell apical and basal structures, which destroys cell polarization, especially apical polarization [31]. In premalignant cells, transforming growth factor-beta (TGF-β) plays a growth inhibition role. So, it is the loss of the tumor inhibition mediated by TGF-β that is an important characteristic of many malignantly transformed cells [39]. PAK4 can weaken the growth inhibition mediated by the TGF-β1/small mother against the decapentaplegic (Smad) pathway, which may cause gastric cancer (GC). Through kinase-independent interaction with Smad2/3, PAK4 blocks the phosphorylation of Smad2 at Ser465/467 or Smad3 at Ser423/425 induced by TGF. Under HGF stimulation, PAK4 phosphorylates Smad2 at Ser465, leading to the degradation of Smad2 through the ubiquitin-proteasome pathway [40]. The oncogenic properties of PAK4 have also been confirmed directly. When iMMECs stably expressing PAK4 were implanted into the breast fat pads of athymic mice, breast tumors were formed in the mice [31].

### 4.2 PAK4 and proliferation

In the process of tumor cell proliferation, PAK4 shows positive significance in regulating cell mitosis [41] and controlling the cell cycle [42]. Inhibition of endogenous PAK4 delays the process of mitosis, and after PAK4 is depleted, the cell even shows mitotic defects such as a lack of centrosome splitting, multipolar spindles, and chromosome lagging [41]. Moreover, PAK4 phosphorylates GTPase Ran at Ser135. The phosphorylation of Ran prevents its binding to Ran guanylate exchange factor (RCC1) and Ran GTPase activating protein (RanGAP1) and inhibits the loading and hydrolysis of GTP at different stages. The result is prevention or maintenance of Ran activation, respectively, which regulates the assembly of Ran-dependent complexes on the mitotic spindle. PAK4 is also closely linked to the regulation of the cell cycle, and it plays a vital role in both the G1 phase and the G2/M transition phase. One study showed that the loss of PAK4 leads to a decrease of G1 cells and induces a block in the G2/M transition, which suggests that PAK4 plays a crucial part in the control of G1 and G2 checkpoints. The level of PAK4 increases transiently in the early stage of G1, and the increase of PAK4 leads to a decrease in P21, which can be a cyclin-dependent kinase inhibitor [42,43]. Similarly, overexpression of PAK4 inhibits the expression of cyclin-dependent kinase inhibitor P57 Kip2 and promotes the degradation of P57 Kip2 through the ubiquitin-proteasome pathway [44]. In addition to the regulation of mitosis and the cell cycle, PAK4 promotes cell proliferation via multiple pathways. Glycometabolism provides energy for tumor cells to support the rapid proliferation of tumors [45]. One of the ways PAK4 promotes proliferation is by promoting glycometabolism. In colon cancer (colon adenocarcinoma, COAD) cells, PAK4 boosts glucose intake and NADPH production, thereby mediating COAD cell proliferation. PAK4 accomplishes this by increasing the activity of glucose-6-phosphate dehydrogenase (G6PD). Interestingly, rather than mediating P53 ubiquitination and degradation directly, PAK4 promotes these processes by enhancing the binding of murine double minute 2 to P53, thereby increasing G6PD activity [46]. The phosphoinositide 3-kinase (PI3K)/protein kinase B (AKT) pathway is crucial in tumor proliferation. In breast cancer (BC) cells, PAK4 activates the PI3K/AKT pathway to promote proliferation [47]. In endometrial cancer (EC) cells, the PI3K/AKT pathway activates PAK4 under the influence of estrogen. Activated PAK4 increases the level of estrogen receptor alpha (ERα), and the higher level of ERα promotes the activation of estrogen-mediated PAK4. The two promote each other to form a positive feedback loop, thus increasing the expression of CyclinD1 and promoting cell cycle progression, prompting the proliferation of EC cells [48]. Pathways such as PAK4/c-Src/epidermal growth factor receptor (EGFR), the interaction of PAK4 with β-catenin, and PAK4/LIM domain kinase 1 (LIMK1)/Cofilin–1 are also important in proliferation. In ovarian cancer (OC) cells, PAK4 controls the expression of CyclinD1 and cell division cycle 25A (CDC25A) by activating the c-Src/EGFR pathway, thereby inducing cell proliferation [49]. Similarly, PAK4 binds and phosphorylates β-catenin, inhibiting the degradation of β-catenin and promoting T-cell factor (TCF)/lymphoid enhancer factor (LEF) transcriptional activity induced by β-catenin. Also, PAK4 nuclear accumulation enhances β-catenin nuclear import, promoting TCF/LEF activity, stimulating the expression of CyclinD1 and c-myc, and leading to cell proliferation [50]. The expression of PAK4/LIMK1/Cofilin–1 increases in osteosarcoma (OS) cells, promoting the proliferation of OS cells [51]. Unlike the two cyclin-dependent pathways mentioned above, Cofilin–1 is an actin-binding protein that may participate in mitosis by regulating the cytoskeleton to promote proliferation itself [52].

### 4.3 PAK4 and survival

Under natural conditions, tumor cells often have tenacious vitality, attributed to their resistance to apoptosis [53] and escape from cell senescence [54]. The resistance of tumor cells to apoptosis is based on inhibiting the apoptotic pathway [55] and activating the survival pathway [56]. In the process of apoptosis inhibition, inhibition of the caspase cascade is key [57]. Overexpression of PAK4 can protect cells from apoptosis induced by serum withdrawal, tumor necrosis factor-alpha (TNFα) treatment, and UV irradiation. It inhibits the proapoptotic protein Bad by enhancing phosphorylation at Ser112 and Ser136, which likely prevents the release of cytochrome C in mitochondria and inhibits activation of caspase–3, thereby inhibiting apoptosis [58]. Also, PAK4 can prevent the caspase cascade by inhibiting the recruitment of caspase–8 to TNFα receptor 1 (TNFR1) [59]. Similarly, PAK4 is essential in the nuclear factor-κB (NF-κB)-mediated survival pathway. PAK4 plays an active role in the TNF-α-induced and NF-κB-mediated survival pathways by promoting TRADD and TNFR1 [60]. PAK4 can also enhance the nuclear accumulation and transcriptional activity of NF-κB by activating Akt and extracellular-signal-regulated protein kinase (ERK) pathways, thereby mediating the activation of cell survival pathways [61]. Cell senescence arrests growth, preventing the development of cancer [62]. Therefore, extended tumor survival cannot be separated from the suppression of senescence. One group found that PAK4 can relieve the senescence-like growth arrest of BC cells by inhibiting the RELB subunit of the NF-κB (RELB) – CCAAT/enhancer-binding protein beta (CEBPB) pathway [63]. Moreover, in primary fibroblasts, PAK4 induces premature cell senescence by activating the ERK pathway and the cell cycle inhibitors P16 INK4 and P19 ARF [64]. It is worth noting that this conflicts with findings that PAK4 resists apoptosis or relieves senescence-like growth inhibition. The effect of PAK4 on cell senescence is still controversial and remains to be explored. Whether PAK4 causes these two contradictory effects due to the difference in the intracellular environments of apoptotic cells, senescent cells, and normal cells is worthy of investigation.

### 4.4 PAK4 and metastasis

Malignant tumors often metastasize, so treatment results are frequently unsatisfactory [65]. At the cellular level, the essence of tumor metastasis lies in the migration and invasion of tumor cells, which are attributed to the weakening of cell adhesion and reconstruction of the actin skeleton [66]. Integrin mediates the adhesion of cells to the extracellular matrix (ECM), which is central to the process of migration and invasion. PAK4 can bind to the proximal cytoplasmic domain of the integrin β5 subunit through the IBD. However, there is no simple physical combination between them. PAK4 binds to and phosphorylates the integrin β5 subunit at Ser759 and Ser762 in a kinase-dependent manner, inducing cell migration [67]. PAK4 mediates cell migration, and integrin mediates cell adhesion. Interestingly, there is a negative feedback loop between PAK4 and integrin instead of an antagonistic effect. The connection between integrin and vitronectin in the ECM activates PAK4, but activated PAK4, in turn, inhibits integrin clustering and integrin-F-actin connection, which destroys the stability of the integrin-mediated adhesion structure, thereby promoting cell metastasis [68]. The focal adhesions connect the ECM and the cytoskeleton [69], and PAK4 can act on Rho GTPase to regulate the decomposition and formation of focal adhesions. One group showed that under HGF stimulation, PAK4 phosphorylates GEF-H1 at Ser885 and inhibits the activity of RhoA mediated by GEF-H1, regulating adhesion. Also, PAK4 promotes the decomposition of focal adhesions by paxillin at Ser272 [70]. Unlike the inhibitory effect of PAK4 on RhoA, PAK4 protects RhoU from ubiquitination in a kinase-independent manner, simultaneously driving adhesion turnover and promoting cell migration [71]. PAK4 plays a crucial part in the regulation of the actin cytoskeleton in addition to the process of adhesion. PAK4 is an effector molecule of Cdc42Hs. Under the induction of the activated form of Cdc42Hs, PAK4 redistributes in the Golgi apparatus, induces filopodia, and initiates local actin polymerization in the Golgi apparatus [27]. PAK4 also interacts with the neural Wiskott-Aldrich syndrome protein (N–WASP) Verprolin Homology/Cofilin/Acidic (VCA) domain. On this basis, PAK4 phosphorylates the N–WASP VCA domain at Ser484/Ser485, increasing the polymerization rate of actin at the forefront and promoting cell migration [72]. Cell invasion requires degradation of ECM, and the actin-rich invasive pseudopodia participate in this process [73]. In the process of invadopodia formation, PAK4 has also shown a promotion effect. PAK4 mediates invadopodia maturation in melanoma cells by inhibiting postsynaptic density protein 95/disc-large/zonula occludens-RhoGEF (PDZ–RhoGEF) [74]. The underlying mechanism may be that Rho has an inhibitory effect on invadopodia, and PDZ–RhoGEF has a Rho activation effect, so the inhibition of PDZ–RhoGEF leads to a decrease in Rho activity, which leads to the formation of invadopodia [75]. In addition to regulating the internal activities of cell metastasis, PAK4 promotes tumor cell migration and invasion via multiple pathways. Of these, the PAK4/LIMK1/Cofilin–1 pathway has been researched relatively extensively. In OS, the PAK4/LIMK1/Cofilin–1 pathway is upregulated, promoting its migration and invasion. In contrast, silencing PAK4 causes pathway inhibition, weakening the migration and invasion ability of OS cells [51]. In GC cells, DiGeorge critical region 6 L (DGCR6L) interacts with PAK4 and enhances the phosphorylation level of LIMK1 and cofilin in a dose-dependent manner, promoting the migration of GC cells [76]. Similarly, HGF stimulates cell migration in prostate cancer cells via the PAK4/LIMK1/Cofilin–1 pathway [77]. Also, the following factors can all mediate migration and invasion: the PAK4/PI3K/Akt pathway, the PAK4/CEBPB/Claudin 4 (CLDN4) pathway, inhibition of P53, activation of superior cervical ganglia 10, enhancement of c-Src and ERK1/2 activity, induction of Matrix metallopeptidase 2 expression, and binding to eukaryotic elongation factor 1 alpha in a kinase-independent manner [49,78,79,80,81,82].

### 4.5 PAK4 and drug resistance

At present, chemotherapy is still the dominant treatment option for cancer, although there are many ways to treat tumors. Unfortunately, during chemotherapy, drug resistance often leads to disappointing therapeutic outcomes [83]. Therefore, research on the mechanism of drug resistance has become very relevant. PAK4 is prominently involved in generating tumor drug resistance via multiple pathways. It can confer cis-diamminedichloroplatinum (CDDP) resistance via activation of the mitogen-activated protein kinase kinase (MEK)/ERK and PI3K/Akt pathways in GC cells [4]. Similarly, PAK4 contributes to CDDP resistance via the PI3K/AKT pathway in cervical cancer (CC) cells [84]. As a predictive marker of gemcitabine (Gem) sensitivity, PAK4 is overexpressed in Gem drug-resistant PC cells, while silencing PAK4 restores Gem sensitivity [85]. In addition to the high expression of PAK4, p-Bad protein content was also shown to increase in drug-resistant PC cells. Importantly, silencing PAK4 by PAK4 and nicotinamide phosphoribosyl-transferase (NAMPT) dual inhibitor KPT–9274 reduces p-Bad, leading to the resensitization of Gem-drug-resistant PC cells. This suggests that the PAK4/p-Bad pathway is a positive factor in developing Gem resistance [86]. A recent study has also demonstrated that the novel PAK4 inhibitor, PAKib, significantly enhances the inhibition of Gem on PC [87].

### 4.6 PAK4 and antitumor immune responses

As a new therapy for tumors, immunotherapy shows great potential. At the present stage, cancer immunotherapy mainly includes programmed death 1 (PD–1)/programmed death-ligand 1(PD–L1) and chimeric antigen receptor T cell-related therapy [88]. PAK4, a target of tumor immune escape, is of high importance to regulating antitumor immunity. The multi-omics analysis of bioinformatics revealed that PAK4 is rich in the “T cell receptor signaling pathway” regulates macrophage polarization through heat shock protein 105 kDa (HSPH1), and significantly impacts neoantigen production and tumor immune regulation [89]. PD–1 is expressed on T cells, whereas PD–L1 is expressed on antigen-presenting cells and cancer cells. The combination of the two can inhibit the antitumor immune-mediated by T cells [5]. Thus, the PD–1/PD–L1 pathway is considered a vital cause of antitumor immunosuppression. Activation of PAK4 improves tumor resistance to PD–1 blockade. PAK4 is overexpressed in melanoma with poor T cell infiltration and facilitates the activation of the WNT/β-catenin pathway [90]. The activated intranuclear β-catenin interacts with TCF/LEF to upregulate the expression of PD–L1 [5]. Furthermore, tumors with genetic PAK4 deletion are more sensitive to PD–1 blockade, and the tumor can completely subside under the action of anti-PD–1 drugs. Likewise, the efficacy of anti-PD–1 treatment was improved as impacted by PAK4 and NAMPT dual inhibitor KPT–9274 [90]. There has been remarkable results of CAR-T therapy for the treatment of hematological tumors (e.g., refractory B cell malignancy) [91]. However, since the tumor microenvironment with abnormal blood vessels inhibits T cell infiltration and activation, CAR-T has no significant effect on solid tumors [92]. PAK4, as a selective regulator of genetic reprogramming and abnormal tumor blood vessels, plays a vital role in tumor resistance to CAR-T. PAK4 improves myocyte enhancer factor 2D to be combined with Zinc finger E-box binding homeobox 1 (ZEB1) promoter and induces the expression of ZEB1, which inhibits claudin-14’s transcription, thus improving vascular permeability. Moreover, PAK4 can induce SLUG expression, downregulate the expression of intercellular adhesion molecule–1/Vascular Cell Adhesion Molecule–1, and reduce the adhesion ability of T cells. Accordingly, in glioblastoma, the inhibition of PAK4 can repair the microenvironment of abnormal tumor blood vessels and increase the efficacy of CAR-T [93].

## 5 Clinical and pathological significance

Although tumor surgery and chemotherapy technologies have developed tremendously, patient prognosis is still unsatisfactory due to tumor metastasis and spread [94,95]. Therefore, identifying tumor biomarkers to evaluate and improve the prognostic effect has become a hot research area. As a popular tumor biomarker, PAK4 plays a vital role in the prognosis of patients with various tumors in the digestive system [33], reproductive system [47], and respiratory system [96]. Exhibiting significant clinical and pathological effects, PAK4 is not only highly expressed in a variety of tumor cells as a possible diagnostic and therapeutic target but also related to clinicopathological indicators such as depth of invasion or tumor size, lymph node metastasis, distant metastasis, tumor stage, histological features, and prognosis (Table 1). Among the digestive system tumors, PAK4 plays a vital role in evaluating clinical and pathological indicators. One study demonstrated that patients with high PAK4 expression in OSCC tend to have poor overall survival (OS) rates and that tumors with high PAK4 expression have a larger volume and deeper invasion depth [33]. In GC, high PAK4 expression is not only associated with deeper invasion depth but also with more severe lymph node metastasis, distant metastasis, advanced tumor stage, and recurrence. Therefore, it is not difficult to understand why the disease-specific survival rate and relapse-free survival (RFS) rate of patients with high PAK4 expression are poor [97]. In colorectal cancer (CRC), high PAK4 expression is closely related to serous layer infiltration and advanced tumor stage but has no connection with lymph node metastasis and disease-free survival (DFS) [98]. Another study showed that high expression of PAK4 in COAD is associated with advanced tumor stage and histological grade, but not with the depth of invasion, lymph node metastasis, or distant metastasis [46]. The fact that high expression of PAK4 is not related to the depth of invasion seems to be inconsistent with the fact that high expression of PAK4 is related to serous layer infiltration; so whether the difference in the scope of inclusion of the two samples (the former was CRC and the latter COAD) or the limited number of samples caused the differences remains to be explored. It is also worth noting that contrary to the poor prognosis associated with high PAK4 expression, for PC patients, PAK4-negativeness is closely related to poor OS and DFS. PAK4-negative PC exhibits larger tumor volume and poor histological differentiation. However, the samples for this study came from patients who may have already been cured, and the number of samples was limited. For the general PC population, the question of whether low PAK4 expression indicates a poor prognosis still needs further investigation [99]. PAK4 has also shown its clinical and pathological importance in reproductive system tumors. In BC, high PAK4 expression usually leads to poor OS and DFS and is closely related to larger tumor volume, more lymph node metastasis, and advanced AJCC stage, but it is not associated with histological grade [47]. Another study showed that PAK4 gradually increased with the progression of BC (advanced invasiveness > early invasiveness > noninvasiveness) [100]. For patients with OC, the activation and overexpression of PAK4 are often associated with poor prognosis. The OS and DFS period of patients with overexpressed PAK4 of OC are shorter. Not only that, PAK4 is linked to the advanced FIGO stage and poor histological grade [49]. Similarly, in CC, high expression of PAK4 is also related to poor OS, lymph node metastasis, distant metastasis, advanced FIGO stage, and poor histological grade [84]. It is interesting to note that low PAK4 expression predicts a poor prognosis for patients with EC. Low PAK4 expression is also closely related to deeper myometrial invasion, advanced FIGO grade, and histological grade. These findings contradict those of the abovementioned studies, namely that high expression of PAK4 is linked to poor prognosis in other reproductive system tumors. The endometrium is a periodic proliferation tissue sensitive to hormone regulation. Whether the contradictory result is related to the unique biological environment of the endometrium is a question worthy of further study [101]. In addition, in non-small cell lung cancer (NSCLC), a respiratory tumor, PAK4 is considered to be an independent predictor of poor survival. NSCLC patients with upregulated PAK4 tend to have a shorter OS period, and high PAK4 expression is closely related to lymph node metastasis, distant metastasis, and advanced tumor stage [96].

**Table 1:** Association between the expression of PAK4 and clinicopathological factors

Tumor	PAK4	T (depth of invasion or tumor size)	N (regional lymph node metastasis)	M (distant metastasis)	Tumor stage	Survival	Histological feature	References
**OSCC**	**+**	T3–4, ***P*** **= 0.0112**	*P* = 0.126	*P* = 0.1052	*P* = 0.1048	Poor OS, ***P*** **= 0.0164**	Differentiated degree, *P* = 0.1486	[33]
**GC**	**+**	T2–4, ***P*** **< 0.001**	N1–3, ***P*** **< 0.001**	M1, ***P*** **< 0.001**	II–IV, ***P*** **< 0.001**	Poor DSS, ***P*** **< 0.001**; Poor RFS, ***P*** **< 0.001**	Differentiated degree, *P* = 0.215	[97]
**CRC**	**+**	T3–4, ***P*** **= 0.012**	*P* = 0.753		III–IV, ***P*** **= 0.004**	Poor DFS, *P* = 0.234	Differentiated degree, *P* = 0.307	[98]
**COAD**	**+**	*P* = 0.656	*P* = 0.425	*P* = 0.543	III–IV (pStage), ***P*** **= 0.02**		Histological grade, ***P*** **= 0.003**	[46]
**BC**	**+**	T2–3, ***P*** **= 0.007**	N2–3(pN), ***P*** **= 0.027**		III (AJCC stage), ***P*** **= 0.012**	Poor OS, ***P*** **= 0.003**; Poor DFS, ***P*** **= 0.001**	Histological grade, *P* = 0.230	[47]
**OC**	**+**				III–IV (FIGO Stage), ***P*** **< 0.05**	Poor OS, ***P*** **< 0.05**; Poor DFS, ***P*** **< 0.05**	G3 (Histological grade), ***P*** **< 0.05**	[49]
**CC**	**+**	*P* = 0.59763	N1, ***P*** **= 0.00525**	M1, ***P*** **= 0.02105**	IIb–IIIb (FIGO Stage), ***P*** **=** 0.00822	Poor OS, ***P*** **= 0.0014**	G3 (Histological grade), ***P*** **= 0.03464**	[84]
**NSCLC**	**+**	*P* = 0.757	N2–3, ***P*** **= 0.034**	M1, ***P*** **= 0.022**	III–IV, ***P*** **= 0.042**	Poor OS, ***P*** **= 0.002**	Differentiated degree, ***P*** **= 0.020**	[96]
**PC**	**-**	**All** T3–4				Poor OS, ***P*** **= 0.003**; Poor DFS, ***P*** **= 0.016**	Differentiated degree, ***P*** **< 0.001**	[99]
**EC**	**-**				III–IV (FIGO Stage), ***P*** **= 0.017**	Poor OS, ***P*** **= 0.026**	G3 (Histological grade), ***P*** **< 0.05**	[101]

OSCC: oral squamous cell carcinoma; GC: gastric cancer; CRC: colorectal cancer; COAD: colon adenocarcinoma (colon cancer); BC: breast cancer; OC: ovarian cancer; CC: cervical cancer. NSCLC: non-small cell lung cancer; PC: pancreatic cancer; EC: endometrial cancer; OS: overall survival; DSS: disease-specific survival; RFS: relapse-free survival; DFS: disease-free survival.

All *p* values in bold mean “<0.05”, indicating significant differences.

## 6 Conclusions

**Figure 3:**
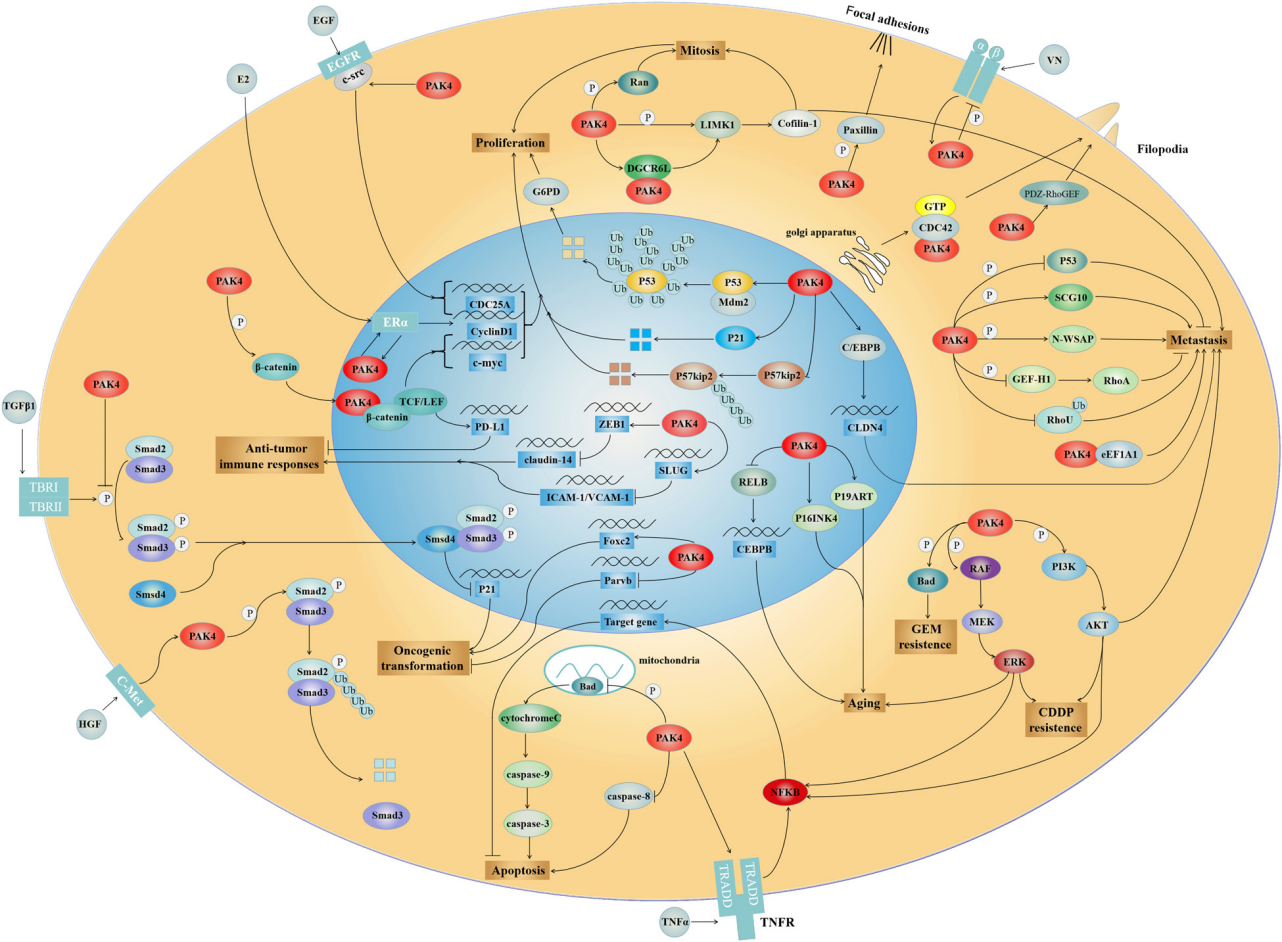
PAK4 signaling pathways. PAK4 promotes cellular proliferation (top). PAK4 regulates cell mitosis to facilitate proliferation by phosphorylating Ran and activating the LIMK1/cofilin–1 pathway. PAK4 improves the binding of Mdm2 with P53 to facilitate the ubiquitination and degradation of P53 and increases G6PD activity. Furthermore, PAK4 promotes ubiquitination degradation of P57 kip2 and P21 degradation. PAK4 and ERα activate each other, thus forming a positive feedback loop to upregulate the expression of CyclinD1. PAK4 activates the c-Src/EGFR pathway and upregulates the expressions of CyclinD1 and CDC25A. In addition, PAK4 phosphorylates β-catenin and enhances its nuclear import. Endonuclear β-catenin and TCF/LEF work jointly to upregulate the expressions of c-myc and CyclinD1. PAK4 promotes cellular metastasis (right). PAK4 interacts with DGCR6L to improve the phosphorylation level of LIMK1 and cofilin. PAK4 facilitates metastasis by activating CEBPB/CLDN4 and PI3K/Akt pathways, inhibiting P53, activating SCG10 and N–WASP, and inhibiting RhoA exchange activity mediated by GEF–H1 in a kinase-dependent manner. Furthermore, PAK4 protects RhoU from ubiquitination while binding to and activating eEF1A1 in a kinase-independent manner. PAK4 phosphorylates the integrin β5 subunit while inhibiting the activity of integrin. The connection between integrin and VN activates PAK4, thus regulating the cell movement through the negative feedback loop. PAK4 phosphorylates paxillin and facilitates the decomposition of adhesive spots. Induced by activated CDC42, PAK4 induces filamentous pseudopodia formation. Moreover, PAK4 inhibits pseudopodia maturation mediated by PDZ-RhoGEF. PAK4 promotes drug resistance and regulates cellular aging (bottom right). Phosphorylation of Bad by PAK4 endows tumors with Gem resistance. Meanwhile, CDDP resistance is imparted via PI3K/Akt and MEK/ERK pathways. In addition, PAK4 inhibits the senescence-like growth arrest of cells mediated by the RELB-C/EBPβ pathway. PAK4 mediates cell senescence by activating the MEK/ERK pathway and the P16 INK4/P19 ARF. PAK4 inhibits cellular apoptosis (bottom). PAK4 inhibits the activity of Bad by phosphorylation. It can prevent the release of cytochrome C in mitochondria and inhibit the activation of caspase–9/Caspase–3. In addition, PAK4 can inhibit apoptosis by inhibiting caspase–8. PAK4 facilitates the binding of TRADD to TNFR1 while inhibiting apoptosis under TNF-α inducement and NF-κB mediation. Moreover, PAK4 activates both PI3K/Akt and MEK/ERK pathways, thus having an effect on NF-κB so as to inhibit apoptosis. PAK4 promotes oncogenic transformation (bottom left). PAK4 interacts with Smad2/3 in a kinase-independent manner to block TGF-β induced phosphorylation of Smad2/3. Besides, PAK4 phosphorylates Smad2 in response to HGF, thus mediating the ubiquitination of Smad2. PAK4 upregulates FoxC2 expression and downregulates ParvB expression. PAK4 inhibits antitumor immunity (left). PAK4 induces the expressions of ZEB1 and SLUG, thus inhibiting the expressions of Claudin–14 and ICAM–1/VCAM–1, respectively. PAK4 upregulates the expression of PD–L1 via the β-catenin pathway. Activation and inhibition of proteins are indicated by arrows and blocked lines, respectively. P: phosphorylation; Ub: ubiquitination; LIMK1: LIM domain kinase 1; Mdm2: murine double minute 2; G6PD: glucose–6-phosphate dehydrogenase; ERα: estrogen receptor alpha; EGFR: epidermal growth factor receptor; CDC25A: cell division cycle 25A; TCF: T-cell factor; LEF: lymphoid enhancer factor; DGCR6L: DiGeorge critical region 6L; CEBPB: CCAAT/enhancer-binding protein beta; CLDN4: Claudin 4; PI3K: phosphoinositide 3-kinase; AKT: protein kinase B; SCG10: superior cervical ganglia 10; N-WASP: neural Wiskott-Aldrich syndrome protein; GEF: guanosine exchange factor; eEF1A1: eukaryotic elongation factor 1 alpha; VN: vitronectin; PDZ-RhoGEF: postsynaptic density protein 95/disc-large/zonula occludens-RhoGEF; Gem: gemcitabine; CDDP: cis-diamminedichloroplatinum; MEK: mitogen-activated protein kinase; ERK: extracellular-signal regulated protein kinase; TNFR1: tumor necrosis factor alpha receptor 1; NF-κB: nuclear factor-κB; TGF-β: transforming growth factor beta; HGF: hepatocyte growth factor; Smad: small mother against decapentaplegic; FoxC2: Forkhead Box C2; ParvB: Parvin Beta; ZEB1: Zinc finger E-box binding homeobox 1; ICAM–1: intercellular adhesion molecule–1; VCAM–1: vascular cell adhesion molecule–1; PD–L1: programmed death ligand 1.

The influence of PAK4 runs throughout the genesis and development of tumors. From preneoplastic cells losing homeostasis to tumor cells with continuous proliferation and from migration and invasion to treatment-resistant tumor cells, complex biological processes and rich signaling involve PAK4 (Figure 3). Activated PAK4 promotes the proliferation [47], migration, invasion [51], and treatment resistance [86,93] of tumor cells through the activation of various signaling pathways. Afterward, these cell activities and characteristics can lead to volume increase, diffusion, metastasis, and reduced sensitivity to chemotherapy and immunotherapy of tumor tissues, because of which the prognosis of tumor patients with high PAK4 expression is often poor. PAK4 is also minimally expressed in normal tissues, while it is overexpressed in various tumors and thus regarded as a tumor marker and treatment target [61]. Also, PAK4 is closely related to tumor infiltration depth, lymph node metastasis, stage classification, and other pathological indicators [97], whose clinical application prospects are broad.

At the moment, PAK4 has been studied extensively in relation to tumor biology function, but there is still conflicting information regarding its role in cell aging. It is contradictory that PAK4 inhibits aging by inhibiting RELB and mediates premature cell aging by activating the ERK pathway and cell cycle inhibitors P16 INK4 and P19 ARF [63,64]. Drug resistance and immune escape of tumors have been reported as the major challenges of chemotherapy and immunotherapy [83,102]. PAK4 resists drugs, and antitumor immune response based on multiple mechanisms, which has been found as a novel target to solve the low efficiency of chemotherapy and immunotherapy [5]. KPT–9274 stands out in targeting PAK4 to improve the efficacy of tumor therapy. KPT–9274 is recognized as a dual inhibitor of PAK4 and NAMPT, which is capable of effectively inhibiting the growth of mantle cell lymphoma, follicular lymphoma, and diffuse large B-cell lymphoma [103]. Moreover, it can significantly improve the efficacy of chemotherapy and immunotherapy in cooperation with GEM or anti-PD-1 treatment [86,90], which can be considered a novel direction in the combined treatment of tumors. However, PAK4 is not the specific target of KPT–9274, and the effect of the inhibition of PAK4 should be studied in depth. Ideal tumor markers need high sensitivity and good specificity. They are also related to tumor size, stage, and severity and can be used to detect tumor localization and efficacy [104,105,106]. Although PAK4 is related to tumor size, stage, and severity, it is overexpressed in various tumors with poor specificity [33,84,96]. Meanwhile, its sensitivity for detecting tumor positioning and tumor efficacy has not been determined. We expect high expression of PAK4 to indicate poor patient prognosis in tumor cases, but in PC [99] and EC [101], contrary to most findings, low PAK4 expression seems to be closely linked with poor prognosis. However, this result was not conclusive and should be confirmed by further research.

To summarize, PAK4 plays an important role in tumor occurrence and development and is important in prognosis and as a tumor biomarker. This article summarizes the role of PAK4 in tumor cytogenesis, proliferation, survival, migration invasion, and drug resistance. We also highlight its clinical pathological significance to benefit preclinical and clinical research on PAK4 and tumors.
